# Cultural Sensitivity in Preventive Infant Mental Health Care: An Example From the Developmental Guidance Project FIRST STEPS in Belgium

**DOI:** 10.3389/fpsyg.2022.831416

**Published:** 2022-05-04

**Authors:** Patrick Meurs, Judith Lebiger-Vogel, Constanze Rickmeyer, Gül Jullian

**Affiliations:** ^1^Sigmund-Freud-Institute, Frankfurt, Germany; ^2^Institute of Educational Sciences, University of Kassel, Kassel, Germany; ^3^Faculty of Psychology and Educational Sciences, KU Leuven, Leuven, Belgium; ^4^Institute of Family Sciences, Odisee University College, Brussels, Belgium; ^5^Paediatrics and Neonatal Care Unit, Brugmann University Hospital, Brussels, Belgium

**Keywords:** developmental guidance, prevention, cultural sensitivity, psychodynamics of migration and exile, applied psychoanalysis, infant mental health care in a context of diversity

## Abstract

Preventive developmental guidance programmes have been applied on a large scale for several decades now in many western countries. But how do we adapt these programs to families with very different ethnic backgrounds? How can we concretise the concept of cultural sensitivity into that context? The plea of von Klitzing, the former President of the World Association for Infant Mental Health, for further reflection on the concretization of *cultural sensitivity* in the context of infant mental health care is the main source of inspiration to this article. von Klitzing speaks out against the point of view in which universal children’s rights or conditions that are seen as promoting a child’s development all around the globe, are criticized as being only western conceptions and thereby culturally biassed. Following this kind of reasoning, a culturally relativistic stance on what a facilitating environment is, should be adopted. Such a discussion in terms of universalism versus relativism, though, as argues von Klitzing, is an old antagonism that cannot inspire us for the adaptation of early health care practices or developmental guidance programs that are fitting in to the needs of families and their babies that are living in multicultural contexts. Although it could be interesting to think about how certain universal principles of good-enough child care can be formulated or embodied in an intercultural variety of ways, these variable expressions of the worldwide agreed upon principles of good early mental health care will only be culture-specific translations of these universal principles. They can’t in any way be seen as an argument against the universality of these principles, as argues von Klitzing, who is describing another way of solving the problem of the cultural relativist criticism on the universality of principles of good infant mental health: *culture sensitive infant mental health services.* But, to make the principle of cultural sensitivity work in practice, we need to fill it with content. This article is mainly on what it concretely means to work in a culture sensitive way in our preventive practice within the FIRST STEPS program, a Belgian project for immigrant and exiled mothers and their children from zero to three.

## Introduction: A Historical Perspective on the Relationship of Cultural Anthropology and Psychology

“Cultural systems of meaning seem to play a crucial role in mediating the relationships between caregiving and child behaviour, suggesting the very real possibilities of a cultural psychology that combines the best of anthropological and psychological methods and disciplines.” (Emde and Spicer, 2000, p. 313).

When we read this quotation, we are repeatedly struck by the cautious wording used by Emde and Spicer in the year 2000, to refer to the role of “culture” in care and child development as well as in prevention science. This caution – “culture seems crucial,” “suggesting possibilities of a combination of the best of psychology and anthropology” – shows that no longer than two decades ago, there was still a long way to go at the level of interaction between anthropology and psychology, before the creative possibilities of culture-sensitive methods within psychology and preventive science could become clear. As mentioned by von Klitzing, very recently, in 2019, a systematic reflection on and description about what it concretely means to work in a culturally sensitive way, still needs to be done.

It has already been more than four decades since Fraiberg et al. (1980) launched the concept of “developmental guidance”:

“*We called this form of treatment “developmental guidance,” in which the therapist becomes a non-didactic educator of the mother as to the physical and emotional needs of her baby and the ways in which a mother can provide these needs. It is guidance in nutrition and in child development.”* (Fraiberg et al., 1980, p. 12).

The practice of care for the physical and emotional needs of the baby, to which Fraiberg et al. (1980) refer, as well as the granting of meaning to the preverbal/bodily communication of these early needs, have ever since received the attention they deserve in prevention science and in psychoanalytically oriented psychology. In the 1980’s, the intention was to gain an insight into certain early developmental factors which had possibly played a role in creating problems that emerge later on, in childhood or in a subsequent phase of life. From the onset of developmental psychopathology (Sroufe and Cicchetti, 1984) this psychoanalytical perspective was introduced in a broader psychological scientific perspective: the study of the earliest mother-child unit within a developmental psychopathology perspective as a part of infant psychiatry and infant mental health care (Beebe and Lachmann, 2014). The early detection of signs of maladaptive development or of development under stress was linked to the idea of developmentally based treatment. Once the possibilities of this kind of intervention became sufficiently known, research into the earliest mother-child relationship was further intensified (see: Zeanah, 2018).

Within the broad scope of psychology, the fields of developmental psychology and developmental psychopathology have devoted much attention to contextual influences on child development (Shonkoff and Meisels, 2000). In studying child development in context, however, the cultural dimension remained one of the least developed contextual aspects, as Emde and Spicer made clear in the year 2000.

For its part, as long ago as the nineteen thirties and forties, *cultural anthropology* pointed to the influence exerted by culture on early care practices and on the effect, which this early care in turn had on the emergence of certain character traits and personality characteristics within a certain culture. The discussion was often held in terms of shared “national characters” or “basic personalities,” which were reproduced within a certain culture through upbringing, without making any reference to the influence of specific sub-cultural differences or of family systems and individual differences in this process. Recently, however, anthropology has embraced a more complex model, partly in response to this criticism. Here, attention is also devoted to the influence which culture as a symbolic meaning system, has on the more individual meanings that are given to social processes and relational dynamics within which the care of children takes place and through which cultural influences are exerted.

Through these evolutions, psychology and anthropology converged: where anthropology came to think of culture as more differentiated by involving systemic (familial contextual) and individual levels in a culture, psychology paid more attention to the cultural, which forms an important dimension of these systemic and individual processes. This conceptual “rapprochement” between psychology and anthropology should now also be translated into practice, including in the field of preventive development guidance.

## Preventive Projects in Infant Mental Health Within a Multicultural World

Sensitivity to the cultural dimension in preventive developmental guidance is of the utmost importance for Western societies today, because they are characterised by increasing multiculturalism as a result of immigration and globalisation.

Many of the children currently participating in preventive developmental guidance programs in the major towns and cities of the Western world, are growing up in cultural or subcultural environments which differ fairly substantially from those of (North American or Western European) middle-class families, mostly from Caucasian origin. During the first years of life, these children from non-Western families have crucial interactions, which are given form and meaning by widely varying cultural environments (Gaskins et al., 2017). These have been brought along within the immigration and keep their influence in the country of arrival. At the same time, these cultural meaning systems of origin are changing, based on their own dynamics of transformation and change as well as in interaction with newly encountered (Western) cultures.

Over the course of the first 3 years of life, intense bodily-affective communication and the initial acquisition of language are, within the context of immigration, substantially influenced by various cultural systems – those of the country of origin and of the country of immigration, for example. Nonetheless, precisely in this phase of life – for reasons we will describe later – the culture of origin of the parents or grandparents of the new-born plays a very crucial role (Moro, 1994). The prenatal phase and the first years of life are particularly suitable for observing the influence and importance of these cultural roots at work in the practices and communication of the parents and grandparents in question, as well as in their internal representations, which form the in-depth psychological foundations of these practices and communications (Moro et al., 2008).

Moreover, this strong influence from the culture of origin during the first years of life certainly applies not only to poorly acculturated or newly arrived mothers, as is sometimes alleged. Even more integrated mothers perceive this phase as a return to or a re-immersion in their “own motherland” in the widest sense of the word. Surprisingly enough, this meaning system that is rooted in the cultural life-world of the land of origin is not often systematically integrated within Western based preventive guidance projects.

The meaning attached to origin and descent also helps define, for example, the identity assigned to the baby at an imaginary level even before its birth within its family and in the wider socio-cultural environment of which that family forms part. In this sense, the imaginary child, the intense preverbal affective communication and the first words exchanged between parents and baby in the first years of life, form the privileged domains for the intergenerational transmission of the cultural life-world of the country of origin from the parents or the grandparents. The transmission of this non-western cultural life-world takes place at the same time as the process of acculturation within these migrated families. A central point in our argument is that, during the first years of life, these families are extremely sensitive to issues such as “roots and integration,” “origin and future,” “continuity and discontinuity” in the cultural identity. In this paper, we will specify how, at the time of the *Motherhood Constellation* (Stern, 1995) and during the period of *Zero to Three*, the sensitivity of parents to their cultural life-world in the land they left as well as their wish to introduce their new-born in a new cultural context, is dealt with over the course of the preventive developmental guidance program FIRST STEPS. An important aspect of valuing cultural life-worlds of origin is the acknowledgment that culture, history and identity that is brought with in the immigration process, are sources of strength and resilience for immigrant and asylum-seeking families, in the process of meeting and integrating newly encountered cultural life-worlds^1^.

## Setting Up Preventive Developmental Guidance for Immigrated Parents and Their Babies in Brussels’ Multicultural Quarters: The First Steps Project

Firstly, we provide some information about the history of the preventive project, implemented originally in “The Families’ House,” a community centre for preventive medical and mental health care in Kuregem, one of the poorest quarters of the Belgian capital, Brussels. Kuregem is predominantly characterised by an immigrant population: currently over 85% of the residents are of foreign origin and this percentage continues to rise.

### A Wide Range of Cultures of Origin

The FIRST STEPS thus was implemented in Belgian districts that have been infamous for generations for their poverty and low social status. The first location of FIRST STEPS in Kuregem-Brussels, is a quarter that attracted immigrants since the end of World War II. The first ones were Italians, Greeks, Portuguese, Spanish and Polish immigrants, soon to be followed, since 1963, by immigrants from other countries around the Mediterranean Sea, all of them so called “guest-workers” from Morocco, Turkey, Algeria, and so on. After 10 or 15 years, some have found success on the socio-economic ladder and left this disadvantaged quarter of Brussels. The places left open by the “leavers” since the beginning of the nineteen eighties have been occupied, on the one hand by new Turkish and Moroccan immigrants coming to Europe for family reunification or family formation/marriage migration, and, on the other hand, by new groups of immigrants coming for political reasons (exiled or dislocated people, asylum-seekers). The latter group of immigrants came from many different directions; Eastern Europeans, specifically from the former Yugoslavia and former Soviet-Union, and immigrants from sub-Saharan Africa, particularly the Congo, a former Belgian colony, Cameroon, Niger, Nigeria, Ethiopia, Eritrea, Sudan and Rwanda, form the largest subgroups among these immigrants. Recently, more Iranian, Iraqi, Syrian, Palestinian, Eritrean, Sudanese and Kurdish asylum-seekers also arrived in Kuregem, as did Afghani families, not infrequently bringing with them a history of suppression, civil war and terror as a result of dictatorship, political instability or far-reaching lawlessness in their countries of origin. In addition to economic immigrants and political refugees, Kuregem is currently also the residence for Romany gipsies, coming chiefly from Slovakia and Romania, where they have been marginalised and threatened with ethnic cleansing since the fall of the communist regimes and the upcoming of nationalists tendencies, or by parts of Turkish or Moroccan families that since 50 years have been living in Southern-Europe and now seek support in North-Western Europe, in the aftermath of the bank crisis in countries like Greece, Spain, Portugal or Italy since 2010.

### Early Developmental Pathways of Immigrant Children: Developmental Vulnerabilities and Their Relationship to Problematic School Trajectories Later on in Childhood and Adolescence

In the past, quarters like Kuregem have often been seriously neglected by national politics and local representatives. Until recently – unless they apply for Belgian nationality and have acquired this several years beforehand – immigrants and asylum seekers had no voting rights and therefore did not seem a priority at political level. This discrimination led to a situation where investment in Kuregem was only forthcoming when serious rioting broke out among adolescents and young adults, who were stuck in particular ghetto schools and/or became unemployed on a large scale during periods of economic crisis or, during economic recovery, could not benefit from the situation. Nonetheless, such outbursts were only the tip of the iceberg: the underlying problems were much broader and occur much earlier in the lives of young immigrants, in the form of developmental problems during childhood (see also: Garcia-Coll, 2012).

From international PISA-Studies (see: Duru-Bellat and Suchaut, 2015), we know that countries like Belgium and Germany have a school system with a rather good outcome, but both countries do not succeed enough in breaking down social inequalities in the course of the schooling period. For example, recent studies on children in immigrant families, starting elementary school (in Belgium at age six) show that they lag behind in terms of social skills, language acquisition and cognitive development (Meurs, 2017). In the early stages of elementary school, a not insubstantial number of these children are also already showing evidence of learning problems and emotional difficulties, while problems of adaptation to the school system clearly increase from the age of ten onward (Nicaise, 2020).

As far as learning difficulties of children with an immigration background are concerned, we see the following in Belgium: by the age of 10, 12% of girls and 21% of boys are already 1 year behind at school, compared to “only” 3 and 5% of middle-class girls and boys without immigration history (Meurs, 2015).

Out of a group of 234 immigrant children (106 boys and 128 girls), born in Kuregem in the year 1995, 21 boys and 53 girls completed General Secondary Education without having to repeat a year in 2013, which is approximately 30% of these children. For middle-class children this number is at 67%. Ten of these twenty-one boys began courses at a college or a university: seven of them completed these courses. Sixteen of the fifty-three girls began studies at this level; fifteen of them also completed them. Of those with an immigration background who did not complete General Secondary Education (160 children; approximately 70% of the 234 children involved in the study), a majority took some form of Vocational Education (Technical Schools), although a considerable number of them, mainly the boys (*N* = 42), did not complete the training, as they became demotivated and dropped out. It is also a known structural problem in Belgium that young immigrants with the slightest academic problem are immediately referred to the lowest educational level, without being spread among the intervening levels (PISA 2015-Study of OECD, 2016). Drop-out in school between 16 and 18 years of age is found by 23% of the youngsters with an immigration background in Kuregem, compared to 7% of youngsters without an immigration background (PISA 2015-Study of OECD, 2016).

The departure from normal academic development paths becomes very clear to many of the teachers and parents involved in the second part of elementary school (at age 9 or 10) and during adolescence, but the process sets in much earlier. However, the early signs of problematic development – in the first year of elementary school or even earlier, at kindergarten and in early childhood – are much less obvious. Good tools for the early detection of these signs are therefore needed. These could be slotted into preventive programs which are aimed at as wide a target group as possible (preventive and community oriented developmental guidance), particularly because this target group as a whole remains a risk group today, even if in the meantime some changes have occurred. The number of children with an immigrant background completing school, for example, has indeed significantly increased, while access to college and university is also higher compared to the previous decades. This does not alter the fact that, even today, the number of children quickly falling behind at school and/or experiencing socio-emotional problems remains disconcertingly high in this and similar districts, certainly when an immigration background is combined with low SES (Meurs, 2015).

The results of our first studies in this respect have encouraged policymakers in Belgium to consider the importance of the early detection of developmental problems and of preventive action. The FIRST STEPS is one of the initiatives that are supported financially in this respect, promoting adaptive early development, thereby preparing children for integration in pre-school at age 3 and in elementary school at age 6. The aim is to offer these children more fair chances in school by preparing the socio-affective, cognitive and linguistic basis for entry in school (see also: Lanfranchi, 2016; Cops et al., 2018).

### FIRST STEPS as a First-Phase Intervention Within a Stepwise Guidance Approach Throughout Childhood and Adolescence

The developmental preventive program FIRST STEPS in Belgium has been built up step by step. The most intensive programs are aimed at parents and their children from zero to three. Parents with an immigration background, most of them with origins around the Mediterranean sea, Minor Asia or sub-Saharan Africa, are mostly not in the habit of systematically placing their children in crèches and with babysitters during the first 3 years of life; they are more likely to look for support for raising children within the wider family context or with grandparents. Care in crèches or with childminders is also mostly difficult to afford for underprivileged parents. The FIRST STEPS, then, like many comparable programs in other countries, is an early opportunity for parents to bring their young child in contact with an extra-familial learning environment, before the child enters pre-school (Lanfranchi and Kohli, 2019).

For parents with pre-school children aged between 3 and 6 years old, a follow-up program – SECOND STEPS – is provided. This involves fortnightly or monthly meetings, which are absolutely essential for consolidating certain effects of the early program. Since children between the ages of 3 and 6 usually go to pre-school, during this period the parents more often come to the meetings without their children. Parenting growing up children becomes an important topic in SECOND STEPS.

Further follow-up is possible within the third step of the stepwise intervention: FURTHER STEPS for parents and children during basic school (6–12 years), and YOUTH STEPS, weekly meetings on Saturdays, for adolescents with an immigrant background, as well as monthly meetings for their parents during evening sessions in the week.

## Methodology of Preventive Developmental Guidance in First Steps

### Three Ports of Entry Into the Early Parent-Infant Relationship

The preventive project FIRST STEPS consists of three sub-projects:

• the First Steps (developmental guidance and educational support project);
• the First Words (language acquisition project);
• “Bon Appétit” (healthy nutrition project).

These three sub-fields – education/development, language and nutrition – serve equally well as windows of entry on the early relational dynamics within which children develop. The counsellors in the three sub-projects therefore devote the majority of their attention to the affectively relational dynamics within which upbringing takes place and language is acquired. If problems arise in one or more of these sub-fields, the affectively relational bond between mother and child during infancy and the toddler years also presents a preferential window for intervention. Emde et al. (2000) describe this kind of interventions as early relationship-based interventions, with the attachment relation as the central working tool for fostering socio-emotional, cognitive and linguistic development.

In order to reach the target group to the greatest extent possible, information is disseminated at various levels. Firstly, the building where the FIRST STEPS project is located also houses the local prenatal and postnatal health care centres run by the two organisations recognised by the Belgian government in this field: *Kind & Gezin* (*Child and Family*) on the Flemish side, *Office de la Naissance et de l’Enfance* (the Office of Birth and Childhood) on the French-speaking side. As a result of this shared location, most of those in the target group – pregnant and new mothers and their partners from this district – come into very early contact with the FIRST STEPS prevention program. Secondly, the developmental counsellors of FIRST STEPS attach the utmost importance to build networks of caregivers and organisations in the quarter the project is running. Thirdly, a weekly presence amounting to half a day in the crèches and nursery schools in the district also means that many parents can become familiar with the project *via* this route. In addition, the developmental counsellors in FIRST STEPS do everything they can to announce their project in General Practitioners’ waiting rooms. Close collaboration with doctors and paediatricians, midwives, nurses, crèches and childminders means that many concerns from parents about the development of their children reach the project counsellors. Fourthly, it is of the greatest importance for the participation of immigrant mothers in the project, that some key figures in their immigrant community become enthusiastic about this project. These key figures are often people who know a lot about concerns and problems within families in the neighbourhood and can refer these families to the project. Fifthly, our developmental guidance fits in closely with certain programs, which have been developed by universities in the countries of origin of these families and implanted in Belgium, such as the Mother and Child Education Programmes (Kagitcibasi, 1996). They often find acceptance among Turkish and Arab immigrants, but are very specifically targeted at five or six-year-old children and are more cognitively learning oriented in comparison to FIRST STEPS with its focus on communicative and affective backgrounds of early social learning processes. Sixthly, FIRST STEPS is part of a larger network of nursing facilities, pre-schools, elementary schools and secondary schools, as well as services for home observation and consultation. In these networks, the nursing and teaching staff members are brought together in two-weekly reflective supervision groups, on themes such as “creating equal chances through developmental guidance and school teaching,” or, “cultural sensitive work with children and parents from immigrant and refugee families.” These reflexion groups for professionals in nursing services and schools are organised by the counsellors and therapists of the FIRST STEPS project, whereas the home consultations are organised by another, autonomously working organisation that is also part of the local prevention network. Seventhly, besides the core business of the FIRST STEPS project (group work with parents and children), there was a possibility for the participants to have individual consultations (over a short period of time in a “five-sessions model”) in order to discuss some very personal questions and problems or to talk about topics that could cause shame and embarrassment in case of disclosure within group meetings in a context where the level of social control can be high.

### Group Work as a Method: Cultural Meanings and Embeddedness of the Gathering of Mothers to Talk About Children and Upbringing

The groups in the developmental guidance sub-project of FIRST STEPS consist of six to eight parents and their children, meeting together every week for 2 to 3 h. There are various types of groups:

• mother/child groups (only mothers and baby);
• mothers’ groups;
• “three generation groups” (with grandparents, parents and child);
• observation and exercise groups (for parents and their child with diagnosed problems, cf. below).

A father/child group was originally also set up but was not able to continue due to work constraints of the fathers that were involved. Yet, as a result of this attempt, a number of fathers did develop the idea of remaining involved with the project in another way: some fathers bring their wives or children to the groups, others stay for the first 15 min of the group session or come 15 min before the end of the group, still others do all kinds of small jobs in the building where the project is organised.

A major source of inspiration for this project comes from the initiative taken by the French psychoanalyst Françoise Dolto in parts of Paris, with the opening of *Les maisons (Ou)vertes*: open houses where parents come to talk about upbringing and development [see: Aubourg (2009), Paglia (2017)]. These houses are designed to be *“des lieux d’accueil et de rencontre”* (spaces of arrival and of being welcomed, spaces for meeting), *“des espaces jeu et loisir”* (spaces for play and leisure) and *“des espaces parole”* (spaces for talking), where parents can talk in the presence of their children (*faire circuler la parole* – pass the word around) and can share questions, concerns, joy and sadness about their children and parenthood – *“des espaces de partage”* (spaces for sharing affective experiences).

In Turkish language, these meetings are referred to by the term *dertlesmek* (the sharing of sorrow and joy, of complex affects and concerns on raising children Devisch and Gailly, 1985). During the meetings in FIRST STEPS, these cultural habits are occasionally evoked: the coming together of mothers thus becomes significant against the background of this cultural heritage; in the meaning which these meetings have for mothers, similarities and differences are perceived between “there and then,” “there and now” and “here and now.” What is it then to raise children here, with a reference to the land of origin from which the grand-parents emigrated?

One important additional aspect of this intervention is that these families are welcomed very explicitly at the beginning of each group session. “Being welcomed” and “being able to participate” is an important theme in the lives of every immigrant. After 3 years of participation in the FIRST STEPS project, a mother of Moroccan origin said that she now knew that “there is something helpful for us, immigrants, here, within this (Belgian) society.” She added that, before participating in the project, she doubted about really being welcome in Belgium. FIRST STEPS made her realise that also the children in immigrants families can obtain support. This mother also became aware that her own perspective on the guest society had changed in a positive way; as she expressed it: “I feel more attached to this society now.” Some of the parents we contacted several years after participation in the program mentioned that their experience of “getting more equal chances and help for their children” was interiorized and guiding them at certain key moments later on, when the developmental course of their children was under pressure. At that moment, they remembered very well that “there is help for them and that they are not left alone with their problems.” This, again, is an important aspect on the way to more equality for the upgrowing children in immigrated families.

### The Meeting Room as an Embodiment of the Linking of Different Cultural Meaning Systems

The surface area of the meeting room for the mother-child groups is ideally approximately between 70 and 100 m^2^. The room is divided into three sections of approximately equal size: one area for meetings between parents, one for children to play and one part for interaction between parents and children.

Chairs are provided *for the parents*, with stools (*tabourets*) in between: low benches, which are customary in countries bordering the Southern and Eastern part of the Mediterranean Sea – the Maghreb countries – and in Asia Minor, since the majority of the participants is from that region. In between the chairs and benches are small, low tables with all kinds of bowls and tea sets. The set-up of the meeting room is in some ways the same as the room in which guests are received in Mediterranean cultures. The small tables can also serve as workbenches for the children, since they are low, if they want to draw, do puzzles or play near their parents. In addition, the meeting room for parents also has corners where children can be cared for or breastfed, if the mothers do not want to do this in the group.

Most of the mothers we meet in the project also spend parts of the summer holidays in their country of origin. They often like to bring back a gift for the project: children’s books, toys or items and ornaments for the meeting room (bowls, tea sets, wall hangings or woven tapestry from the countries of origin of these families) are extremely welcome, as well as fruit and water which has been brought back from “over there” and is generously shared with the group in the first session after the holidays. In this way, an intercultural mix is gradually produced, in the available toys and in the furnishing of the meeting room, which reflects the widely diverse origins of the participants in the groups as well as the culture of the country where the project is running.

Several play areas are provided *for the children*, equipped with toys suited specifically to various ages between 0 and 6. Each child has an individual drawer (as it is common in psychoanalytic child psychotherapy) where all kinds of drawings can be kept, as well as clay models, things they have made themselves or brought along. The toys include a lot of dolls, as well as human figures with widely varying clothing and racial features. Furthermore, there is also a wide range of animals, drawing materials, building blocks, construction material, cars, a farm, a garage, a kitchen, a doll’s house, a castle, etc. Activity sets and Maxi-Cosi strollers are provided for the smallest children. The room also has a wall cabinet with children’s books in various languages, as well as books without text. Reading books together or reading children’s books aloud is an important activity for mothers and children together; illiterate mothers – who initially avoided the book corner – can also get involved in telling stories, using the books without text. Reading in front of children is also a means to immerse them in language(s).

Between the play areas for the children and the meeting room for the parents – where parents and children are often also present together – is a *transitional room for interaction between parents and children*. This is particularly where the mother/child bond (attachment?) during the first 2 years of life is practised. This is the area where *floortime* (Davis et al., 2014) takes place: explicit attention is devoted to bringing about and maintaining communication between parent and baby. Stimulated by the “developmental counsellor,” the parents learn to look at interaction with their children in a new way, in order to recognise the positive aspects more explicitly and to reinforce them. The intermediate space where this subtle interactional game takes place is separated from the rest of the room by a low plastic tape. Parents and children can go back and forth around this border, between the meeting space for the adults and the play areas for the children, without disrupting mother/child interaction during the floortime.

### The Developmental Counsellors as Facilitating Environmental Figures, Symbolising Diversity Within a Multi-Lingual Social Context

The team of the FIRST STEPS project consists of one male and two female care workers, one of Belgian origin and the others with immigrant backgrounds themselves. The three counsellors are clinical psychologists, social workers or educational scientists with a different ethnic background. They are trained as psychoanalytical child psychotherapists and/or systemic supervisors. Furthermore, the project also involves translators or intercultural mediators, particularly for parents from Eastern European, Minor Asian or Sub-Saharan backgrounds.

The topics brought up for discussion during the group sessions are not fixed in advance. The participants define them jointly. The counsellor is present and fulfils the functions primarily of “welcoming” and “containing” (holding difficult and/or intense affective experiences, giving meaning and communicating this meaning for affects that otherwise are overwhelming or unbearable). He/She facilitates the coming up of questions about education, child development and parenting. In this sense, he/she monitors the “potential space” for meeting each other and talking to each other around these topics. The counsellor repeatedly reminds the group that it meets each week at a fixed time and that certain matters which could not be fully discussed can be brought up again the next time.

The counsellors also switch between the various subgroups in the meeting room or circulate between parents and children. In the presence of the children, the counsellor attaches much importance to structuring the play activities. Parents take over this aspect themselves in time; they are strongly encouraged to do so, as is the case with the managing of conflict situations between children over the use of toys. These incidents are preferably discussed in the mothers’ group afterward, where much importance is attached to comparable conflicts in the home situation and the roles of fathers and mothers in such situations.

## Making Culture More Concrete in the Context of Developmental Guidance and Infant Mental Health Care

### Attachment to Culture of Origin and the Wish for Integration Within Newly Encountered Cultural Life-Worlds

Immigration is not a subject in itself in this project. At the same time, immigration plays an extremely important role in virtually all aspects of education and development, so that this subject is always implicitly or explicitly present in the stories the mothers tell and in the questions they ask.

Grinberg and Grinberg (1989) describe immigration as a far-reaching change, more specifically as a loss of cultural self-evidence. It is not culture that is lost, it is the self-evidence of a cultural meaning system that is called into question. Immigrants can process this loss in a creative way; in anthropological literature, the results of this processing have been described as the emergence of a bi-cultural (LaFramboise et al., 1993) or twin identity (Werbner and Modood, 1997), a multiple (Littlewood, 2000) or mixed identity (Akin, 2002). These concepts from the acculturation literature and research (see: Berry et al., 2002, 2016) make very clear that a process of coming to a creative bi- of multicultural identity does not exclude conflicts along that process and that in the end bi- or multicultural identity is at the same time attachment to origins and orientation toward integration (Meurs, 2017). In that process the richness of bicultural belonging is acknowledged, while at the same time tensions and conflictual aspects of bicultural belonging are endured. The less creative processing is expressed, on the one hand, in an obstinate clinging to everything from there-and-then, which impedes any change in cultural identity. On the other hand, it is all too easy to be absorbed in a new cultural identity, without leaving room for putting down roots in the culture of origin. Both figures of less creative identity finding in a multicultural environment are characterised by a reduction of the multiple into the singular.

### Realising That Specific Waves of Immigration Are Associated With Specific Challenges, Vulnerabilities as Well as Elements of Resilience (Dynamics of Economic Migration, Political Exile, Transnational Migration)

A key moment arises in the family cycle with the birth of a new generation, when even the most creative balance between continuity and discontinuity in cultural identity is itself again brought into question. Among parents, for example, the question remains of how they will introduce both cultures to their children. We regard this as a general issue associated with the immigration process.

In addition, a more specific question emerges: that of the impact on the parent-child relationship of the specific waves of immigration from Mediterranean cultures. This immigration took place in stages or shifts. From the beginning of the 1960s onward, Western European countries relied heavily on immigrants from Southern Europe, North Africa and Asia Minor to fill particular vacancies in heavy industry (mining, steel). This first generation of immigrants was therefore made up of men. They came with the idea of re-emigrating: earning good money over a few years in Western Europe, then building a better life in their country of origin, using the financial and material gains from immigration. This remigration was repeatedly postponed as a result of the healthy economic situation in the 1960s and early 1970s. Only during in the summer months did these men often return to their families in their country of origin for a couple of weeks.

However, things changed with the economic crisis of 1973. One year later, in 1974, a ban on immigration – “the migration-stop” – was announced throughout Western Europe; immigrants could now only come in order to reunite families. This led to a feminisation of immigration, when the spouses of the first generation of immigrants made use of this opportunity to support their husbands’ immigration projects. Those already in Western Europe no longer remigrated; after all, it was no longer certain that anyone going back would have a second chance at economic immigration to Western Europe.

Around 1975, the spouses of the guest workers had in many cases become mothers in their country of origin, before they came to Europe. When they emigrated, they too did not intend to stay here long and, with this in mind, not seldom left their first-born children with grandparents or aunts, to be raised there, among family members in the country or origin. The idea was to leave the parents here with their hands free and to put their efforts into work for a further short period. Moreover, leaving the children at home meant a counterbalance for the breaches and discontinuities, which were experienced by both immigrants and those who stayed behind in the homeland. Leaving the children there during their early years was a visible sign that all concerned were convinced that the whole family would be together again there soon. For some years, the first generation of immigrants in western Europe – both men and women – lived with the idea that they would re-migrate within the foreseeable future and, at that point, would slot back in neatly with their own parents and children in the region of origin.

However, from the beginning of the nineteen eighties, it became clear that the migration-stop would not be lifted in the short term. Anyone who did nonetheless re-emigrate was faced with a dilemma: on the one hand, as an economic migrant he/she could not then return to Western Europe, on the other hand – as a re-immigrant with more than a decade of absence – he/she was not always openly welcomed back at home. In the meanwhile, between 1980 and 1990, other children were born in the families of guest workers in Western Europe and were raised in a European context from birth on. As a result of these macro-economic circumstances and the evolutions of perspectives on migration within the community of origin of these immigrants, over the course of the nineteen eighties, immigration took on a more permanent character. In response to this, mothers in particular encouraged the older children to be sent over, which again was made possible with a view to family reunification. These children, raised in the country of origin in the first years of life, as well as their brothers and sisters subsequently born here, are referred to in the literature as “the second generation of immigrants.” The vast majority of the parents we have welcomed between 2000 and 2015 in the FIRST STEPS project in Belgium belong to this generation. With the so-called second generation and their children that have been born from the end of the 20th century on, immigrants became an ever more permanent part of the population in western European countries and the question of whether these co-citizens can be labelled any further as immigrants is justifiable, since most of them are born and have been raised in western countries. The grand-parents (the first-generation immigrants) – men in particular – still talk of going back, but these remigration projects are increasingly being called into question as the children and grandchildren grow up in Europe and are of course Europeans themselves. Ultimately, also most parents of the first generation remain with their (grand)-children in Europe.

In the years that went by since 2000, we could often hear that the position of the young men and women of the second-generation is not easy in Turkish and Moroccan immigrant communities. Since the ban on immigration was never lifted, considerable pressure is exerted on the young men and women in these communities to marry someone from the country of origin of the (grand)parents. Nowadays, “importing brides and bridegrooms” is a variation on the practice of family reunification: only by marrying someone from Turkey or Morocco can young men or women from these countries, who are not part of the European Union, come to Western Europe in a legal way. Family reunification has thus become active family formation or marital migration, which again allows the ban on immigration to be evaded. Sociological research indicates that around 2010 approximately 50% of marriages in Turkish immigrant communities currently take place in this way (Caestecker et al., 2018).

Second-generation marriages usually take place during summer holidays in the country of the emigrating partner. These marriages can be recognised by western European juridical systems. Then, the spouse can request entry into Europe as a form of family reunification. However, what is regarded from a Western perspective as family reunification is a highly complex reality at affective-relational level. Marriages with “imported partners” produce parent couples with widely varying socialisation histories, which can occasionally make it more difficult to raise the next generation. A good knowledge on this kind of mostly economic migration to Europe and on the specific challenges that are associated to this kind of migration is important for a better understanding of the marital and educational questions that can arise in these families.

A woman from a Turkish or Moroccan family who has grown up in Western Europe and who marries an imported bridegroom often has to “fend for herself” during the first years of the marriage. While the bridegroom tries to settle down here to some extent, begins to learn the language and often has to work for some time below his level of education and, moreover, before they can afford themselves their own house, is living with his mother-in-law, the woman often is the major breadwinner, the most efficient bridge between family and the outside world and, at the same time, is (traditionally) responsible for bringing up the children. If a man who grew up here in an immigrant family “imports” a bride, the woman must often go to live with her mother-in-law for some time. This may well be in accordance with specific common traditions in this area, as is the case in Central Turkish culture, but nonetheless this tradition does involve certain risks in the context of immigration. While the imported bride tries to adapt to some extent, she not infrequently feels suddenly cut off from her own relatives, who now live a few thousand kilometres away. Feelings of loneliness are often no stranger to these imported brides. In this difficult situation, such women not infrequently become first-time mothers.

Luyckx (1998) demonstrates that psychological and relational difficulties are far from infrequent in these marriages. And it is no coincidence that this group, mostly aged between 20 and 30, is proportionally over-represented in residential infant psychiatry wards in Flanders to which both mothers and new-borns can be admitted. There, they regularly represent over 20% of the patient population, with a postpartum pathology of a depressive and/or psychotic nature (Meurs, 2015).

Moreover, the age at which the young immigrant women we met in the Belgian FIRST STEPS project come to marry, is significantly lower than that of other women becoming mothers for the first time. The lower marriage age, by Western standards, could indeed be inherent in the cultures of origin, but forms a risk factor in an immigration context. The social support from the extended family is by no means as self-evident as it is within the culture of origin. Immigration has after all spread extended family members throughout Western Europe. So also raising children in an immigration context is more often characterised by loneliness and emotional strains for these young mothers.

### Adopting a Multigenerational Perspective on Immigrated or Exiled Parents and Their Babies

The cultural change caused by immigration has brought about certain socio-emotional vulnerabilities. The young mothers of the second generation often describe that they find insufficient identification possibilities in their mothers of the first generation. With respect to the dialogue between cultures, first and second generation mothers adopt a different attitude. This is partly a result of the fact that the first generation of mothers from the 1970s to the 1990s did not come to Europe until they were on average over thirty years old, while the second generation had been here since childhood or birth. The mothers in the second generation describe how they look elsewhere for identification figures, particularly among contemporaries in age: sisters, cousins, girlfriends. However, here too, the feeling often arises of being left to one’s own devices: the impression is of being charged with the same task as other women of the same age, but still of having to find one’s own individual answer. Moreover, second-generation mothers often have the impression that they would like to impart to their children aspects of the cultural background which they received from their parents, but that their children of the third generation will inevitably be very different from them, so that they do not know if they are doing right or wrong in raising their children in the way they do. Second-generation mothers thus often feel that they are on their own, both inter-generationally and intra-generationally.

A point of identification between immigrant mothers of both generations emerges when the young mothers of the second generation realise that they are living with the same fear as their parents – specifically, the fear that their children will be different, that their children will not consider their parents in their quest for an identification figure. This is expressed, for example, in a high degree of uncertainty and indecision when the young parents have to make decisions concerning choice of school, choice of language, structuring and letting go of their children, etc. In conversations in the presence of the grandparents, during sessions in the three-generation group, the young parents of the second generation recognise, sooner or later, that these fears were just as evident in the previous generation, among their parents. Discussing this transgenerational transfer of concerns about the upbringing and development of children in a context of immigration and of the impact these fears and concerns can have on parents and children, makes the group meeting into an important transgenerational event for those involved.

The fathers of the first generation were highly focused on remigrating and, in that respect, did not have the intention of showing their children or their wives ways to the broader Western society. However, as soon as they realised that migration had become more permanent, it was not so easy for them to change course. The second-generation fathers are not infrequently just as insecure, either because some of them dropped out from school in Western Europe or because, as imported bridegrooms, they have just arrived from their country of origin and it takes them some years to find their own way in a different cultural environment. The insecurities of immigrant fathers are often discussed in the groups in the context of all kinds of upbringing issues. Particularly in their attitudes toward the school trajectory of their children, the choice of educational language, the representation of authority within the family, these young immigrant or second generation fathers’ insecurities are brought sharply into focus. In the parent/child groups and in the three-generation groups, extra attention is sometimes devoted to the strengthening of the father’s position in the family.

An additional element that needs to be mentioned in this context is the discontinuity that is experienced in the context of migratory processes. Most of the families in FIRST STEPS are part of extended family networks that are dispersed over several European or other countries and that, as a result of this dispersion, have become transnational families. The families we meet in FIRST STEPS mostly present themselves as nuclear families, very similar to what most families have become in the western world. But in the context of the FIRST STEPS meetings, we soon found out that the other fractions of the familial network have an important influence on decisions that have to be made about child development. In that sense, our experiences in the project were also an opportunity to enlarge the ethnocentric point of view of our counsellors, working from expectations that have originated in the context of western nuclear families. A three generations perspective or an extended family or transnational family perspective was often present in the reflections of the parents within FIRST STEPS groups. Like several other aspects that are described in this article, these practice-oriented reflections are closely linked to the theoretical discussion we opened up from the beginning of this article: how can we combine, in a practice-oriented and at the same time theoretical way, culture-specific and universal perspectives on child care and parenting?

### Working With Differing Cultural Scripts Regarding the Emergence and Resolution of Mental Health Problems

A great deal of consultation takes place at times of illness, insecurity and doubt and, equally, at key moments in the family cycle (for example, developmental transitions into adolescence) as well as at times of crisis (for example, a divorce between the parents). In some cases, simultaneous consultations take place with Western care workers and healers from the cultural tradition of origin, for example the *hoça* for Turks, the *fqih* for Moroccans, the *marabout* for western Africans. These co-consultations are described in anthropological literature as medical pluralism (Devisch, 2002) or therapeutic hybridisation (Littlewood, 2002).

The parents in question do not talk very often about this simultaneous consultation in two therapeutic systems. The mothers are by and large more likely than the fathers to invest in this dual practice, but major differences also exist among mothers. Sometimes they explicitly say, “I am doing now what my mother did; I do not know if what she did is right, but nor do I know if it would be right for me to do it differently.” Let us suppose that, even if a mother of Turkish or Moroccan origin does not really believe in it, she would not want to say that she had not sought all possible means of protection or cure for her child.

The significance of this co-consultation does of course differ from parent to parent but, fairly often, the recurrent theme in stories of these consultations is related to concerns regarding the vulnerability of a pregnancy and labour; concern too about what can happen postpartum; concern about vulnerability of babies and their parents and about being negatively influenced by invisible forces.

Differing explanations for illness are put forward at such times of vulnerability, such as the *djinna* or the evil eye (*‘ain*). In contrast to these, we find ritual healing practices, or *türbes* may be visited (shrines for praying for the protective presence of certain ancestors). The consultations with *hodjas*, *fqihs* or *marabouts* and visits to the *türbe* mostly take place back at home, during the summer holidays, but the healers can also be visited all year round, in large Western towns and cities.

The parents in question almost always think a great deal of the problem can be resolved by the Western medical system. However, they also admit that this simply cannot eliminate all existential insecurity or ontological vulnerability for which they feel their culture provides an additional answer.

In preventive developmental guidance, this presents us with culture-specific explanations about the emergence and course of illness, as well as about healing and the role of therapists. In Magrebian cultures, for example, mothers can believe strongly in the evil eye, which is often associated with bad, suspicious, jealous or angry others who will not grant them a healthy baby. The suspect is often someone completely unknown or a relative of someone with whom the mother in question or her family does not get along; this person is supposed to have cast an evil look to bring disaster or sickness upon the child. This explanation binds together the fears for the uncertain or the indefinable, which is strongly felt at times when a child is unexpectedly seriously ill for a long time. This explanation for illness also offers a way out because it always provides an answer: the mothers read passages from the Koran for example, recite fragments of religious texts, carry out certain instructions and place protective objects by the child and near themselves, rituals that go back in time into the pre-Islamic period.

The task of the grandmother at such times is often crucial and placed within a broader social context: she will try to see how the social background to the problem (for example, an outstanding debt, rejected marriage proposal, etc.) can be resolved or reconciliation can be achieved. Where the mother is focused more on her descendants, the grandmother will try to bring about resolution within the extended family, the broader social network of the immigrant community or even the group of origin who remained behind in the country of origin. Sometimes, these beliefs and ritual practices are religiously influenced, in other occasions they are part of local folk medicine. For the counsellors within the prevention project, it is important to have some knowledge on such culture-based explanations and practices as the ones described.

### The Meeting Room as a Potential Space for Weaving Cultural Differences Into a Containing and Diverse Meaning System

By discussing these kinds of practices, the meeting room of FIRST STEPS embodies multiple cultural meaning systems, that of the cultures of origin, Western culture as well as the intercultural collision. The influence exerted by these three aspects of the cultural identity of the participants (with an immigration background) on education and development is repeatedly and explicitly discussed. At times of insecurity and ambivalent fluctuation between different cultural influences, care is taken to ensure that “the intercultural” or the cultural collision does not have a confusing or debilitating effect for the parents and children involved or that these parents and children do not fall *between* two stools (cultures) (see also: Berry et al., 2016). In other words, in this culture-sensitive developmental guidance project, the intention is to maintain a potential space in which the participants can explore aspects of different cultural systems and then incorporate them into a supportive and meaningful system of meanings and practices, while the areas of conflict or the irreconcilability of the intercultural collision can also be discussed. However, oases of creative interculturality do occur when the parents feel they have been helped by the counsellors in recognising the support, which their own cultural background continues to provide at times of change or crisis and in moments of intercultural dialogue. Once the desire for weaving threads to the culture of origin is recognised, they can enter more contentedly into dialogue with other cultures in their environment. With every step they take toward familiarisation with the cultural difference, many of them will again want to strengthen their roots in the cultural tradition that has come along with the generations before them: attachment to culture of origin and a desire to integrate newly encountered culture are both important dynamics in these young families with an immigrant background.

Particularly during the period of imminent new life in the womb or after the birth, with the child at home, at the breast, in the home, at the hearth or at the table, the influence of the culture handed down by the grandparents is particularly strong. Mothers not seldom speak to their babies in Turkish or Moroccan, care for their babies as their parents taught them, prepare meals which are only served in this well-defined traditional way, during this period of *motherhood constellation* and, alongside what the doctor has prescribed, use all kinds of things passed on by grandmother to mother. These traditional motherhood practices are most strongly supported by the counsellors in FIRST STEPS: they form a supportive matrix within which musings are possible about the future transformations in cultural identity of their children and of themselves, once the children go to school. Particularly in the second year of life, when not seldom breastfeeding stops, thoughts occur more frequently about cultural change processes. These thoughts become even more explicit if, during the third year of life, the child’s first steps outside the house – to school or temporary day-care – are prepared for in the project. Quite often in this phase we also find that parents want to teach their children another language – in Belgium: Dutch or French – which they will need during the forthcoming developmental stages. This choice leads to ambivalence: the parents know that it enhances their children’s developmental chances but are also afraid that, precisely as a result, the children could become strangers in the family. At these times too, parents and counsellors together look for ways in which the “culture or language” of the child’s ancestors can remain present within the family. Once this is sufficiently guaranteed, we see that many parents will give their children more room to learn another language and that they quite often go in search of language courses for themselves too, both generations enjoying the acculturation processes within their own family.

## Conclusion

From the beginning of our project FIRST STEPS in the year 2000, culture-sensitive infant mental health work interested us a lot. We soon found out, however, that at that moment the concept of cultural sensitivity was still vague. Ever since, we came to concretize cultural sensitivity in several ways, for example by understanding parent-infant group meetings with immigrant families within the context of activities of parents and babies in the cultures of origin (f.e.: *“dertlesmek”*), or, by dressing the meeting room with objects, ornaments, play toys, children’s books and furniture from different origins, or, by working with a divers team that embodies very different origins and speaks several languages. We felt that these adaptations were important; yet culture-sensitive work requires more than only these transformations.

Another way of working in a cultural sensitive way is to value differences in cultural beliefs or perspectives, for example on what a child is and on how parenting is done, or, on the understanding of educational and developmental problems and on the kind of therapy or healing they need. What parents recount in the meeting groups of FIRST STEPS is understood as an attempt to weave cultural life-worlds (that of origin and the newly encountered one) into a containing bi- or multicultural meaning system, thereby expressing attachment to origin as well as a wish for acculturation and integration. By doing so, the parents try to combine the best of both cultural life-worlds in an attempt to do as much as possible on behalf of the health of their offspring.

This part of the culture sensitive work is about bringing together different local or culture-specific ways of understanding children’s development, in an attempt to rebuild a meaning system in the context of migration: a kind of *bricolage* (Levi-Strauss, 1962), *métissage* (Moro, 1994) or *assemblage* (Marcus and Saka, 2006). The need for such a *bricolage* is connected to what Moro (1994) sees as a metacultural theory: counsellors need to be aware that all over the world cultures have in common that they offer meaning and that they function as containment, and that in the context of migration, cultural meanings systems lose their self-evidence but don’t get lost and can be re-discovered and transformed or adapted to new socio-cultural environments. In the context of immigration one needs to re-weave different and specific cultural perspectives into a containing (bi- or multicultural) meaning system, with all the creativity as well as the conflicts, confusion or even moments of impossibilities within the intercultural encounter this can lead to.

To work with these cultural specificities means that we grant them an equivalent meaning on the symbolic level, but at the same time that these culture specific perspectives are not more or less than emanations of universal aspects and principles of early parent-child relationships.

Culture provides meaning and containment all over the world (a global aspect of culture in general, described in a metacultural theory); the way this meaning and containment is concretized, embodied or brought into practice in daily life can be quite specific in the context of different cultures (a local perspective on specific cultures, described in an intercultural approach). Although the culture-specific expressions are an emanation of the universal function of culture, this universal function of culture can only be realised through culture-specific expressions.

To conclude we can say that, in order to work in a culture-sensitive way within prevention projects, it is important to emphasise the profound relationship between the global and the local perspective on culture, or, between the universal and the relativistic perspectives within cultural theories. As an example of this, we can refer to the given that – universally – cultural meaning systems offer a framework within which to raise children and that migration can lead to profound uncertainties about the culture-specific understanding of the parent-child relationship. Both the meaning system of origin as well as the newly encountered meaning system can feel uncertain within migration. In the prevention group of FIRST STEPS a lot of work is done to re-weave these different culture-specific perspectives into a containing inter-cultural meaning system. Concretely, this also means that culture-specific traditions and meaning systems of the immigrant families – for example about mother-child bodily contact, about breast feeding or sleeping practices – are seen as enriching and equal perspectives or as practices that create a continuing sense of hold and grip within the new context they immigrated to. In that sense, FIRST STEPS is not conceptualised as a one-way help for immigrant families with acculturation problems; the project is rather seen as a mutual learning process where the developmental counsellors too come to value culturally rooted and culturally different perspectives on becoming a parent as well as on raising children^2^.

The search of von Klitzing (2019) for a content of cultural sensitivity has now been resolved insofar as we have been able to leave behind the sterile critique of cultural relativism on universalism (seeing cultural relativism and universalism as complete opposites and rejecting any comparative perspective on non-western and western cultures) as well as the ethnocentric point of view of some universalists (seeing western cultural scripts as self-evident, thereby implicitly considering the western as the universal). We exceeded this sterile opposition by combining the best of cultural anthropology and of cultural psychology. This in turn, made it possible to find answers to Emde and Spicer’s (2000) quest for a integration of cultural sciences within infant mental health care projects that are suited for the needs of today’s superdiverse world.

## Author Contributions

All authors listed have made a substantial, direct, and intellectual contribution to the work, and approved it for publication.

## Conflict of Interest

The authors declare that the research was conducted in the absence of any commercial or financial relationships that could be construed as a potential conflict of interest.

## Publisher’s Note

All claims expressed in this article are solely those of the authors and do not necessarily represent those of their affiliated organizations, or those of the publisher, the editors and the reviewers. Any product that may be evaluated in this article, or claim that may be made by its manufacturer, is not guaranteed or endorsed by the publisher.

## Footnotes

1 Valuing the cultural life-world, identity and history of families and ethnic communities that have immigrated from non-western to western societies and/or from rural and mostly under-served areas to urban contexts with more psychosocial and mental health services, is an important principle in the projects of the Centre of Excellence for Infant and Early Childhood Mental Health Consultation too (IECMHC, 2021).

2 The guiding principles of the FIRST STEPS project are tightly linked to the ten practice-based principles for effective early childhood mental health consultation services, such as they have been described by IECMHC (Centre of Excellence for Infant and Early Childhood Mental Health Consultation) and WAIMH (Word Association for Infant Mental Health): (1) relationship-based, (2) collaboration, (3) individualised, (4) culturally and linguistically responsive, (5) grounded in developmental knowledge, (6) evidence-informed [see: Meurs and Luyten (2006)], (7) data-driven (for example, in continuously data gathering on long-term developmental courses of participating children, (8) delivered in natural settings, (9) a continuum from promotion to intervention, (10) integrated with community services and support (See: Kaufmann et al., 2012).
